# Four priorities for new links between conservation science and accounting research

**DOI:** 10.1111/cobi.13254

**Published:** 2018-11-27

**Authors:** Clément Feger, Laurent Mermet, Bhaskar Vira, Prue F.E. Addison, Richard Barker, Frank Birkin, John Burns, Stuart Cooper, Denis Couvet, Thomas Cuckston, Gretchen C. Daily, Colin Dey, Louise Gallagher, Rosemary Hails, Stephen Jollands, Georgina Mace, Emily Mckenzie, Markus Milne, Paolo Quattrone, Alexandre Rambaud, Shona Russell, Marta Santamaria, William J. Sutherland

**Affiliations:** ^1^ Montpellier Research in Management (MRM) Univ Montpellier Univ Paul Valéry Montpellier 3, Univ Perpignan Via Domitia Montpellier France; ^2^ AgroParisTech 75005 Paris France; ^3^ Centre for Ecology and Sciences of Conservation (CESCO) Museum National d'Histoire Naturelle 43 rue Buffon CP 135 75005 Paris France; ^4^ Department of Geography University of Cambridge The David Attenborough Building, Pembroke Street Cambridge CB2 3QZ U.K.; ^5^ Department of Zoology, Interdisciplinary Centre for Conservation Science University of Oxford Oxford OX1 3PS U.K.; ^6^ Saïd Business School University of Oxford Park End Street Oxford OX1 1 HP U.K.; ^7^ Management School University of Sheffield Conduit Road Sheffield S10 1FL U.K.; ^8^ Business School University of Exeter Rennes Drive Exeter EX4 4PU U.K.; ^9^ Department of Accounting and Finance University of Bristol Senate House, Tyndall Avenue Bristol BS8 1TH U.K.; ^10^ Aston Business School Birmingham B4 7ET U.K.; ^11^ Natural Capital Project, Center for Conservation Biology (Department of Biology), and Woods Institute for the Environment Stanford University 371 Serra Mall Stanford CA 94305 U.S.A.; ^12^ Department of Accounting and Finance University of Stirling Stirling FK9 4LA Scotland U.K.; ^13^ Institute of Environmental Sciences University of Geneva 66 Boulevard Carl‐Vogt 1205 Geneva Switzerland; ^14^ National Trust Kemble Drive Swindon SN2 2NA U.K.; ^15^ Centre for Biodiversity and Environment Research University College London Gower Street London WC1E 6BT U.K.; ^16^ WWF‐UK The Living Planet Centre Brewery Road Woking GU21 4LL U.K.; ^17^ School of Business and Economics University of Canterbury Private Bag 4800 Christchurch 8140 New Zealand; ^18^ The University of Edinburgh Business School 29 Buccleuch Pl Edinburgh EH8 9JS U.K.; ^19^ CIRED, AgroParisTech, Cirad, CNRS, EHESS, Ecole des Ponts ParisTech Université Paris‐Saclay 94130 Nogent‐sur‐Marne France; ^20^ CNRS, UMR, DRM, M‐Lab Université Paris‐Dauphine, PSL Research University 75016 Paris France; ^21^ School of Management University of St Andrews The Gateway, North Haugh St Andrews KY16 9RJ U.K.; ^22^ Natural Capital Coalition 1 Moorgate Place London EC2R 6EA U.K.; ^23^ Department of Zoology University of Cambridge The David Attenborough Building, Pembroke Street Cambridge CB2 3QZ U.K.

## Abstract

*Article impact statement*: New collaborations with accounting research can improve conservation impact of ecosystem‐based information systems.

## Introduction

Engagement with diverse social science disciplines is essential to revealing political, social, and institutional challenges that must be addressed to advance effective biodiversity conservation (Bennett et al. 2017; Teel et al. 2018). One challenge that remains insufficiently investigated is frustration with the lack of impact of innovative information tools and systems of accounts aimed at motivating and guiding ecosystem management. The conservation community invests considerable efforts in their creation and experimentation. Species and ecosystem accounts (e.g., ABoS 2015; UNEP‐WCMC 2016), general ecological indicators (e.g., Jørgensen et al. 2013), and tools for ecosystem‐services quantification and mapping (e.g., Kareiva et al. 2011) and ecosystem monitoring are fundamental to conservation research and practice. However, ecosystem‐based tools do not always lead to the changes in decision, action, or policy conservation scientists expect (e.g., Ruckelshaus et al. 2015).

Often, the inability of such information systems to generate expected changes is not due to technical limitations rather than the too fragile articulation between their design and the complex realities of developing strategies and organizing management of ecosystems in a diversity of contexts. Investigating such articulation between an information system and the organizational details of its systematic use is precisely what characterizes an academic field: accounting, which belongs to management as a discipline and often intersects with social sciences or economics. Accounting has enormous but untapped potential to contribute to conservation science, practice, and goals. Accounting is often misconceived as being only the craft of producing quantitative and financially focused reports for companies. However, accounting in its broadest sense is the preparation and the framing of information (qualitative and quantitative) to assist specific organizing and decision‐making processes (Jollands 2017).

We especially refer here to critical and interpretive accounting research, a field that emerged in the 1970s and developed through now well‐established journals (i.e., *Accounting, Organizations and Society*, *Accounting Auditing and Accountability Journal*, *Critical Perspectives on Accounting*) (Miller & Power 2013; Roslender 2017). Since 1990s, researchers have revealed and criticized the lack of consideration of sustainability issues in existing accounting systems (e.g., Milne 1996) and advocated the development of new accounting approaches inspired by ecological thinking at and beyond the corporate level (e.g., Birkin 1996; Bebbington & Larrinaga 2014; Russell et al. 2017).

Following a recent publication that proposes a new line of inquiry focusing on developing accounting research at the ecosystem management level (Feger & Mermet 2017), a workshop furthered in‐depth interdisciplinary dialogue between accounting scholars and conservation researchers and practitioners. Its results underline that collaboration between conservation and accounting research is essential to improve the design and the actual use of ecosystem‐based information systems for accountable conservation decisions and actions. Four key areas for future joint research were identified.

## What Accounting Brings to Conservation

Our call to establish new links between the accounting discipline and biodiversity conservation is not meant to be a substitute for economics, game theory, organizational psychology, or any other discipline focusing on decision making. It is an invitation to focus on questions instrumental and common to both conservation and accounting research, such as the following: How are records kept in practice and with what consequences? What languages and representations can one provide to complex organizations? Who gives and demands what kind of accounts? How are responsibilities negotiated, organized, managed, and controlled? How are explicit principles and conventions on which accounts can be developed and values defined and on which past and future actions can be assessed and compared debated and institutionalized?

The pervasive confusion in the environmental field between the disciplines of accounting and economics deserves a special comment. Although economics and accounting are somewhat related, they are essentially different disciplines (Shiozawa 1999). Accounting is concerned with developing and using calculative practices to support decision making as is economics. The use of economics in conservation science has brought major results, considering, for instance, the development of economic valuation of ecosystem services, analysis of environmental trade‐offs, and study of incentive structures (Helm & Hepburn 2012). One of the distinctive characteristics of accounting, however, is that it focuses on the detailed analysis of the roles of information systems in the context of the concrete complexities of organizational management based on the fundamental concepts of accounts and accountability (Burchell et al. 1980; Roberts & Scapens 1985; Gray et al. 2014). In terms of methods, accounting research combines theoretical developments that extensively draw on other social science disciplines (organizational theory, sociology, philosophy, economics, psychology, etc.) with in‐depth qualitative field studies of organizations (Ahrens & Chapman 2006). In doing so, it enriches understanding of the role of information systems and accounts in the operationalization of action and generation of intended or unintended organizational changes and wider governance transformations (Miller 2001; Macintosh & Quattrone 2010).

The new dialogue we advocate between conservation scientists and accounting researchers can build on a small but growing body of work in accounting research, centered on ecosystems, that aims to study the effects of varying forms of accounting on relations between human organizations and biodiversity (e.g., Tregidga 2013; Dey & Russell 2014; Cuckston 2017) and develop accounting innovations adapted to the collective management of ecosystems (Feger & Mermet 2017).

## Priorities for Development of Accounting for Ecosystem Management

### Studying Ecosystem‐Centered Accountabilities

A first priority is to study in depth how, in diverse ecosystem management situations, stakeholders actually use or could use ecological and related social, health, economic, and financial information to assign responsibilities to one another and to discuss, negotiate, and manage reciprocal commitments (i.e., accountabilities) for improving environmental outcomes. This means exploring questions such as what commitments have been, are being, or should be negotiated among stakeholders; who is accountable to whom and who is not regarding management of ecosystem quality; and how should information be framed and exchanged to organize these accountabilities effectively? An accounting lens can illuminate how different ways of structuring, representing, giving, and demanding environmental information can lead to creation of viable forms of ecosystem‐centered management to achieve conservation goals (Roberts & Scapens 1985; Dey & Russell 2014; Cuckston 2017; Feger & Mermet 2017).

### Working Collaboratively on Real‐World Cases

Conservation scientists and accounting researchers need to jointly conduct in‐depth studies and comparisons of real‐world field cases through an accounting lens. Thus, a portfolio of case studies reflecting on past cases and observing and documenting active on‐going cases (e.g., through action research interventions) need development.

### Adopting a Constructive, Practical, Critical, and Reflective Approach

In working collaboratively on concrete cases, conservation scientists, accounting researchers, and decision makers will engage in constructive discussion to improve the design and use of ecosystem‐based information tools. This calls for pragmatic trial‐and‐error approaches that rely on action‐oriented agendas and reflexive cultures that are common to conservation science (e.g., adaptive management [Gunderson & Holling 2002] or evidence‐based conservation [Sutherland et al. 2004]) and accounting research (Gray 2002).

### Developing a Common Language

These 4 priority goals require intensive interdisciplinary dialogue and the development of a common language. Accounting concepts need to be adapted and enriched to analyze and discuss the organizing of ecosystem management and conservation action (e.g., *ecological account*, *accounting entities*, *accounting perimeters*, and *accountabilities*) (Russell et al. 2017). The specificities of accounting concepts, as distinct from concepts used in the field of economics or ecology, need theoretical clarification, especially when terms overlap (e.g., *valuation* and *capital*) (Rambaud & Richard 2015). Finally, the formulation of new concepts and vocabularies (e.g., *reciprocal commitments*) has to be central to joint efforts of accountants and conservation scientists to develop an accounting approach for the management of ecosystems.

## Conclusion

The 4 priorities for the development of accounting approaches centered on management of ecosystems set up an agenda that can reshape conservation practice and the way ecosystem‐based information tools are designed and used in conservation and accounting research and the way accounting entities and accountabilities are understood. By collaboration and engagement across these disciplines, there is scope for contributing to constructive critical reasoning and to introduce innovative designs that combine insights from accounting and conservation. Ultimately, this new interdisciplinary bridge will provide a critical, theoretical, and practical addition to the already well‐established collaborations of conservation research with other social science fields, such as economics, anthropology, and political ecology.
